# Acute Hypoxia Stress-Induced Apoptosis in Gill of Japanese Flounder (*Paralichthys olivaceus*) by Modulating the *Epas1/Bad* Pathway

**DOI:** 10.3390/biology11111656

**Published:** 2022-11-12

**Authors:** Guangling Li, Binghua Liu, Jun Yang, Xiaohui Li, Hao Wang, Haishen Wen, Feng He

**Affiliations:** Key Laboratory of Mariculture, Ministry of Education, Ocean University of China, Qingdao 266000, China

**Keywords:** hypoxia stress, apoptosis, *EPAS1/Bad* pathway, DNA methylation

## Abstract

**Simple Summary:**

Hypoxia is perhaps of the most pressing global environmental challenge in aquatic systems. Hypoxic stress can cause physiological changes in fish, resulting in injury and even death to them. Japanese flounder is a crucial benthic economic fish, but the mechanisms of its response to hypoxia are poorly understood. Here, we show that Japanese flounder can alter gill morphology, and induce apoptosis to weaken the risk caused by hypoxic stress. The mechanism of the *EPAS1/Bad* signal pathway in response to hypoxia was investigated from the perspective of transcription and epigenetics. Therefore, our results would enrich the theory of hypoxic stress on Japanese flounder and provide a reference for its healthy cultivation.

**Abstract:**

The physiological responses and molecular mechanisms of apoptosis in Japanese flounder under hypoxic stress remain unclear. In the present study, we performed acute hypoxia stress on Japanese flounder (2.39 ± 0.84 mg/L) and detected gills responses in histomorphology and molecular mechanisms. The results showed that the volume of the interlamellar cell mass decreased and the gill lamellae prolonged, indicating the expansion of the respiratory surface area. Additionally, the fluorescence signal of apoptosis increased under hypoxic stress. In addition, the expression of two genes (*EPAS1* and *Bad*) related to apoptosis increased about four-fold and two-fold, respectively, at 6 h of hypoxia. Meanwhile, the result of the dual-luciferase reporter assay showed that EPAS1 is a transcription factor, which could regulate *(p <* 0.05) the expression of the *Bad* gene, and we identified the binding site of EPAS1 was the AATGGAAAC sequence located near −766. DNA methylation assay showed that hypoxia affected the methylation status of CpG islands of *EPAS1* and *Bad* genes. All results indicated that hypoxia could activate the *EPAS1/Bad* signal pathway to induce gill apoptosis of Japanese flounder. Our study provides new light on understanding the molecular mechanism of hypoxia-induced apoptosis in Japanese flounder.

## 1. Introduction

Hypoxia influences an ever-increasing number of marine organisms as a result of the combined effects of increased global warming and higher nutrient enrichment of coastal waters [1]. Organisms in the marine ecosystem living in anoxic environments, especially fish, are facing unprecedented challenges in development, physiology, and pathology [2]. Fish gills acted as an important role in physiological functions including gaseous exchange, osmoregulation, acid–base regulation, excretion of nitrogenous waste, and hormone production [3,4]. Gills are also the main organs that respond to the change in dissolved oxygen in the aquatic environment [5]. It was reported that the morphological structures of gills in many fish showed significant changes during hypoxia [6]. Hypoxia-induced gill remodeling may be related to changes in the expression levels of apoptosis-related genes [7,8]. Therefore, hypoxia can induce gill morphology changes and cell apoptosis in fish gills.

Hypoxia is a kind of cell stress, and the apoptosis induced by hypoxia may mediate cell damage and even death [9]. Bcl-2 family proteins are important members of the apoptosis signaling pathway. Because the Bcl-2 family can induce apoptosis by releasing proapoptotic proteins, including cytochrome C, into the cytoplasm [10]. *Bad* is one of the major pro-apoptotic genes in the BCL-2 family [11]. The apoptotic function of *Bad* is largely related to the phosphorylation and dephosphorylation of Ser112, Ser136, and Ser155 [12]. In response to apoptotic stimulation, *Bad* is rapidly dephosphorylated and interacts with anti-apoptotic proteins *BCL-2* and *BCL-XL* and promotes cell death [13]. In cells without external stimulation, *Bad* phosphorylates at these sites and forms complexes with 14-3-3 proteins in the cytoplasm, thereby promoting cell survival [14].

*EPAS1* (*HIF2α*) is a member of the HIF family [15]. It is well established that HIFs are essential for responses to hypoxia, which mainly rely on oxygen to control a lot of the expression of target genes. Under normoxic conditions, the degradation of the *HIFα* rapidly happened because of the ubiquitin–proteasome system. In contrast, *HIFα* accumulated rapidly under exposure to hypoxia [16]. *HIFα* can directly or indirectly affect the activation of a range of target genes in hypoxia through epigenetic regulation [17]. *EPAS1* plays a key modulatory role in numerous biological mechanisms. *Hif2α (EPAS1)* and RAS synergistically promote the occurrence of lung tumorigenesis in mice [18]. As a transcription factor, EPAS1 is indispensable in angiogenesis. *EPAS1* may promote mature angiogenesis by transactivating vascular endothelial growth factor (*VEGF*), fetal liver kinase-1 (*Flk-1*), and *Tie2* promoters [19]. It is reported that *EPAS1* can also be negatively regulated by the small non-coding RNA mir-182-5p to promote the invasion and metastasis of lung cancer [20]. In particular, *EPAS1* is associated with apoptosis. *EPAS1* could enhance Fas-mediated chondrocyte apoptosis [21]. Additionally, *EPAS1* and *HIF-1α* may influence neuronal cell apoptosis by regulating the expression of the *BNIP3* gene [22]. However, the role of *EPAS1* in apoptosis through epigenetic regulation has not yet been fully understood.

DNA methylation is an epigenetic mechanism generally related to transcriptional silencing. In some cases, DNA methylation can activate gene transcription [23]. DNA methylation is a covalent modification catalyzed by DNA methyltransferase (DNMT). Additionally, ten-eleven translocation (TET) enzymes may remove active DNA methylation. In addition, the cells contain proteins that recognize DNA methylation (such as the methyl-CpG-binding domain (MBD) proteins, the ubiquitin-like, containing PHD and RING finger domain (UHRF) proteins, and the zinc-finger proteins). DNA methylation in different genomic regions (such as intergenic regions, CpG islands, and gene bodies) may have different effects on gene expression. Notably, the methylation state of CpG islands can destroy the binding of transcription factors and their target genes, and then stably inhibit gene expression [24]. In this article, we examined the methylation status of *EPAS1* and *Bad* promoters and looked for the possibility that they might affect their own gene expression.

Japanese flounder is widely cultured in China because of its excellent flavor, high protein, low fat, low calorie, and rapid growth [25], but very few studies have focused on the apoptosis of the gills under hypoxia. Our previous studies found that hypoxia affects immune-related signal pathways, such as the *STAT3/VEGFA* pathway [26], and *Egr2-FasL-Fas* pathway [27] of Japanese flounder muscle, and the antioxidant metabolic pathway *Keap1/Nrf2 (Mafs)-GST* [28]. In conclusion, hypoxia severely affected the immune signal pathway and antioxidant metabolic of Japanese flounder muscle by changing epigenetic modifications and transcription factors. In the present study, we described branchial histological structure, apoptosis of the gills, and the regulatory mechanism of the *Bad* gene expression by *EPAS1* in the gill of Japanese flounder. Additionally, we decided to investigate if DNA methylation is involved in hypoxia-induced *EPAS1* and *Bad* expression. Our results indicate that the *EPAS1/Bad* pathway is essential to the apoptosis of Japanese flounder gills under hypoxia.

## 2. Materials and Methods

### 2.1. Animal Housing and Hypoxia Stress

The Japanese flounder used in this study were obtained from Qingdao HaoRuiYuan aquaculture Co., Ltd., China. The fish were reared and sampled at Lab of Fish Breeding Physiology and Seed Engineering (Qingdao, China). Firstly, 60 individuals were temporarily housed in three tanks (1.5 m × 1.5 m × 0.6 m) in a recirculating seawater system for 7 days. The water temperature was nearly 16.09 ± 0.89 °C, pH was 7.0–7.5, and dissolved oxygen was 9.48 ± 0.42 mg/L. The photoperiod was 14 h light and 10 h dark. All fish were fed on Microbound Diet (SHENGSUO, Yantai, China) twice a day (8:30–17:30). The contents of crude protein, crude fat, crude ash, crude fiber, calcium, total phosphorus, and lysine in the Microbound Diet are 50%, 7%, 17%, 4%, 5%, 1.2%, and 2.5%, respectively. We removed residual feed and feces 30 minutes after feeding. All fish fasted for three days before the hypoxic treatment.

After all fish fasted for three days, we regulated the water hypoxia (2.39 ± 0.84 mg/L) by pumping nitrogen into the water using nitrogen cylinders. During this period, dissolved oxygen meter was used to monitor dissolved oxygen in water. In the hypoxic treatment, temperature, pH, and photoperiod were the same as in the temporary culture stage. We collected the control group under normal oxygen and the treatment group under hypoxia. The concentration of dissolved oxygen in the treatment group is 2 mg/L, which is more than half the dissolved oxygen concentration [29]. Then, gills of 6 fish were collected after 0 h (control group), 1 h, 3 h, 6 h, 12 h, and 24 h of hypoxia treatment. In detail, firstly, we used MS222 (100 mg/L) to anesthetize Japanese flounder. Secondly, we measured the length (14.92 ± 0.98 cm) and weight (66.59 ± 1.50 g) of Japanese flounder. Then, we dissected the fish immediately, and cut the first pair of gills on the left for branchial histological structure analysis of gills, and the second pair of gills on the left for apoptosis analysis. Both samples were placed in 4% paraformaldehyde. Finally, we collected the remaining two pairs of gills on the left of the fish as samples for DNA extraction, and all four pairs of gills on the right were collected as samples for RNA extraction. The samples for DNA and RNA extraction were placed in liquid nitrogen for quick freezing and then stored in −80 °C freezer.

### 2.2. Branchial Histological Structure

Gills were fixed with paraformaldehyde for 6 h and subsequently transferred to 75% ethanol. Then, gills were dehydrated in an ethanol series (75%, 85%, 90%, 95%, and 100%), rendered transparent with xylene, and embedded in paraffin. Samples were sectioned to a thickness of 5 μm using a microtome (LEICA TP-1020, Wetzlar, Germany) Then, tissue sections were hydrated by ethanol series (100%, 95%, 80%, 70%, and 50%). After standard hematoxylin and eosin (HE) staining of the sections, the slides were sealed with neutral gum (Yi Yang Instrument Co., LTD., Shanghai, China), and the sections were photographed under a microscope (OLYMPUS, Tokyo, Japan).

### 2.3. Apoptosis Detection by TUNEL

For the detection of apoptosis, TUNEL staining was conducted using the TransDetect^®^ In Situ Fluorescein TUNEL Cell Apoptosis Detection Kit (Trans, Beijing, China). The gills were sectioned as described in the 2.2 branchial histological structure. Following the instruction, the paraffin sections were dehydrated in an ethanol series (95%, 90%, 85%, 75%, and 50%), permeabilized, labeled, and photographed under a fluorescence microscope (ECHO RVL-100-G, San Diego, CA, USA).

### 2.4. Phylogenetic Analysis

The amino acid sequences of *EPAS1* and *Bad* gene were obtained from the National Center for Biotechnology Information (NCBI) (https://www.ncbi.nlm.nih.gov/, accessed on 28 August 2022). We used the neighbor-joining method (based on 1000 bootstrap replicates) to construct the phylogenetic trees of *EPAS1* and *Bad* genes. Evolutionary distances were estimated according to the model of Poisson correction method. In the evolutionary tree of *EPAS1*, we used two vertebrates, *Gallus gallus* and *Homo sapiens*, as outgroups. In the evolutionary tree of *Bad*, we chose *Homo sapiens*, *Capra hircus*, *Sus scrofa*, *Chelonia mydas*, and *Xenopus tropicalis* as the outgroup.

### 2.5. RNA Extraction and Gene Expression

Total RNA of gill samples was obtained using Total RNA Extraction Reagent (Vazyme, Nanjing, China). The integrity of total RNA was determined by agarose gel electrophoresis (1.2%), and the concentration of total RNA was assessed with spectrophotometric analysis (A260:280 nm ratio). The cDNA synthesis was performed by HiScript III RT SuperMix for qPCR (+gDNA wiper) (Vazyme, Nanjing, China). Three biological replicates were performed at each time point (0 h, 1 h, 3 h, 6 h, 12 h, and 24 h) under three technical replicates. For qPCR, we used ChamQ™ SYBR^®^ Color qPCR Master Mix (Vazyme, Nanjing, China). Moreover, qPCR used an Applied Biosystems StepOne Plus Real-Time PCR System (Applied Biosystems, Waltham, MA, USA) to complete quantification. The specific primers of *EPAS1* and *Bad* genes were designed by Primer Premier 5 and listed in Table S1. The primer of 18S (as a reference gene) refers to Huang et al., 2019 [30]. The reaction system and procedure of quantitative real-time PCR are listed in Tables S2 and S3. The melt curves of primers for three genes *EPAS1*, *Bad*, and 18S in qPCR were shown in Figure S1. The target gene expression was calculated with the 2^−ΔΔCt^ method [31].

### 2.6. Dual-Luciferase Reporter Assay

We used TF (transcription factors) prediction website HumanTFDB (http://bioinfo.life.hust.edu.cn/, accessed on 28 August 2022) to analyze the promoter of *Bad* gene and found that EPAS1 is one of its transcription factors (Figure S2). The expression plasmid (pc3.1~*EPAS1*) and reporter plasmid (pGL~*Bad*) were constructed by dual-luciferase reporter assay, and we verified the targeting relationship of *EPAS1* and *Bad*. The gills’ genomic DNA was extracted and a 2013-bp fragment of the 5’ flanking region of *Bad* gene was isolated by PCR using 2 × Phanta Max Master Mix (Dye Plus) (Vazyme, Nanjing, China). The PCR product was purified and double digested by KpnI (NEB, Ipswich, MA, USA) and HindIII (NEB, Ipswich, MA, USA) before being inserted into pGL3-Basic plasmid by homologous recombination and this plasmid (pGL~*Bad*) was transferred into DH5α cells (Vazyme, Nanjing, China). Similarly, the *EPAS1* coding sequence (CDS) was obtained by high-fidelity PCR. Then, double digested by BamHI (NEB, Ipswich, MA, USA) and Xhol (NEB, Ipswich, MA, USA) before being inserted into pcDNA 3.1(+) plasmid by homologous recombination, and the plasmid (pc3.1~*EPAS1*) was transferred into DH5α cells. The reaction system and procedure of the double digestion are listed in Table S4. After *EPAS1* and *Bad* sequences were successfully cloned and sequenced (Sangon Biotech, Shanghai, China), plasmids were extracted by using EndoFree Mini Plasmid Kit II (TIANGEN, Beijing, China). Then, we transfected the expression plasmid (pc3.1~*EPAS1*) and reporter plasmid (pGL~*Bad*) into human embryonic kidney 293T (HEK293T) cells by Lipofectamine™ 3000 Reagent (Invitrogen, Waltham, MA, USA). The Renilla luciferase plasmid (pRL-TK) (Promega, Madison, WI, USA) could express Ranilla luciferase. So, pRL-TK plasmid (as a control) was used to quantify the transfection efficiency. Transfection steps were as follows: First, seeded cells were 50–70% confluent at transfection. Second, Lipofectamine™ 3000 was diluted using DMEM/High Glucose culture medium (Servicebio, Wuhan, China). Third, the plasmids were diluted using DMEM/High Glucose culture medium, and the mixture was mixed by adding P3000™ Reagent. Fourth, the second and third products were mixed, and the mixture was incubated for 15 minutes. Fifth, the fourth mixture was added to the cells. Sixth, the transfected cells were analyzed after incubation at 37 °C for 2 days.

HEK293T cells were cultured in DMEM/High Glucose culture medium containing 10% fetal bovine serum (FBS) (absin, Shanghai, China) at 37 °C. HEK293T cells were seeded in 24 well plates (Corning, New York, NY, USA) at a density of 1 × 10^5^ cells per well and incubated at 37 °C. When the confluence of cells reached 50% to 70%, plasmids were transfected into HEK293T cells. Three duplicate wells were set for each group. In the end, double-fluorescence values were detected using SYNERGY HTX multi-mode reader (BioTek, Winooski, VT, USA) and Dual-Luciferase kit (Promega, Madison, WI, USA) after transfection for 2 days (48 h).

To determine the specific binding site of *EPAS1*, we used fragment deletion to narrow the range of binding sites, and then use bases mutation to determine the binding sites. The binding sites of EPAS1 transcription factor on *Bad* promoter sequence were predicted by the transcription factor prediction website. The method of constructing plasmids is similar to that of constructing pGL~*Bad* plasmids. Cell culture, transfection, and determination of double-fluorescence values are the same as the above methods. Fragment deletion plasmids were constructed including pGL~Bf1, pGL~Bf2, pGL~Bf3, pGL~Bf4, and pGL~Bf5. Then, pGL~Bmp3, pGL~Bm3, pGL~Bmp5, and pGL~Bm5 were directed bases mutation reporter plasmids. Fragment deletion and bases mutation details were found in 3.5. EPAS1 transcriptionally regulated *Bad*. The specific primers were designed by Primer Premier 5 and listed in Table S5. The reaction system and procedure of the PCR are listed in Tables S6 and S7. In particular, inverse PCR was used for base mutant plasmids.

### 2.7. Detection of DNA Methylation Level

Due to the *EPAS1* and *Bad* gene expression, four groups (0 h, 1 h, 6 h, and 24 h) were chosen for their promoter methylation analysis. The genomic DNA of the gill was isolated using FastPure^®^ Cell/Tissue DNA Isolation Mini Kit (Vazyme, Nanjing, China). An amount of 1µg of genomic DNA was modified by EpiArt™ DNA Methylation Bisulfite Kit (Vazyme, Nanjing, China). Methylation-specific PCR was conducted by using 2 × EpiArt™ HS Taq Master Mix Kit (Vazyme, Nanjing, China). The prediction of CpG islands and the design of primers were completed by MethPrimer design program (http://www.urogene.org/methprimer/, accessed on 28 August 2022). MS-PCR products of *EPAS1* and *Bad* were cloned into the pCE2 TA/Blunt-Zero Vector (Vazyme, Nanjing, China) to construct plasmids and then the plasmids were transferred into DH5α cells. Three biological replicates were performed in each group and the sequence of 10 clones for each individual has been performed using M13 forward and reverse primers. The formula [30] used to calculate the bisulfite modification efficiency was listed here: (the number of converted cytosines (except cytosines of CpG dinucleotides)/total number of cytosines (except cytosines of CpG dinucleotides) × 100). The specific primers were designed by Primer Premier 5 and listed in Table S8.

### 2.8. Statistical Analysis

All data were represented as means ± standard error. All data were analyzed by one-way analysis of variance (ANOVA) using SPSS 22.0 (SPSS, Chicago, IL, USA), followed by Duncan’s post hoc test. Statistical significance was chosen at *p* < 0.05.

## 3. Results

### 3.1. Effects of Hypoxia Stress on Gill Morphology

**Figure 1:**
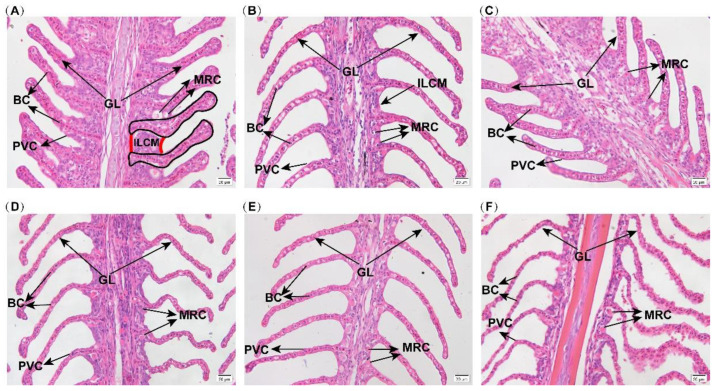
LM micrographs of the gill under hypoxia. The pictures are 0 hours of control (**A**), and 1 hours (**B**), 3 hours (**C**), 6 hours (**D**), 12 hours (**E**), 24 hours (**F**) of hypoxia. The length of the scale bar of (**A**–**F**) represents 20 μm. GL: gill lamellae; BC: blood cell; PVC: pavement cell; MRC: mitochondria rich cell; ILCM: interlamellar cell mass.

In the hypoxia treatment, most Japanese flounder were inactive and had difficulty in breathing. Some fish swam to the surface at intervals to take in oxygen and slowly swam back to the bottom of the tank. Thus, the gills, as respiratory organs, were first examined for morphological changes. HE staining of gill results are illustrated in Figure 1. The gill was neatly arranged at 0 h. With the prolongation of hypoxia time, the volume of the interlamellar cell mass (ILCM) decreased and the gill lamellae elongated, which increased the area of gill lamellae contact with water. Additionally, the gills became curved after 6 h and 24 h of hypoxia. The exposure to hypoxia triggered morphology change in the gill.

### 3.2. Effects of Hypoxia Stress on Gill Apoptosis

**Figure 2:**
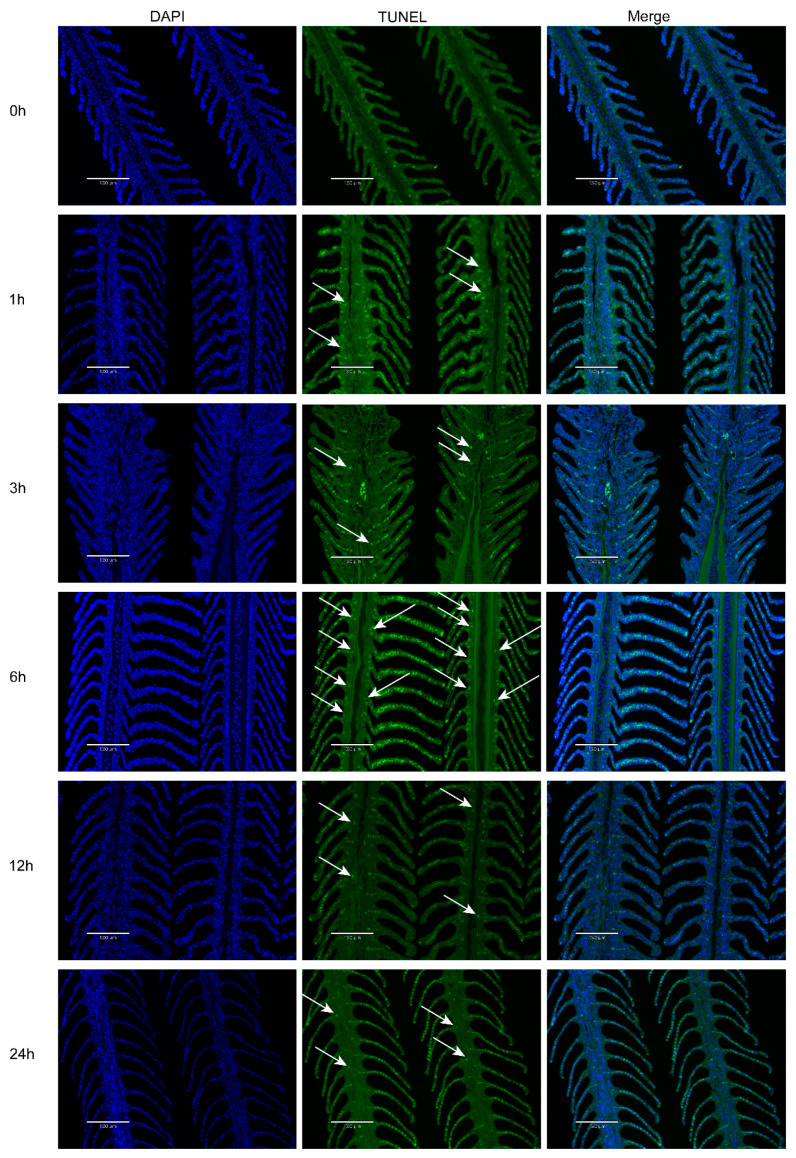
Apoptosis of gills under hypoxia. The gills of apoptosis showed green fluorescence and times on the left showed hypoxia stress times. The length of the scale bar represents 130 μm. The arrows in the figure point to the location of the apoptosis signal.

There was almost no TUNEL apoptotic signal in the 0 h group. However, the TUNEL apoptotic signals increased with the extension of hypoxia time. In detail, the apoptotic signals began to appear at 1 h of hypoxia and were very dense at 6 h of hypoxia (Figure 2).

### 3.3. Phylogenetic Analysis

**Figure 3:**
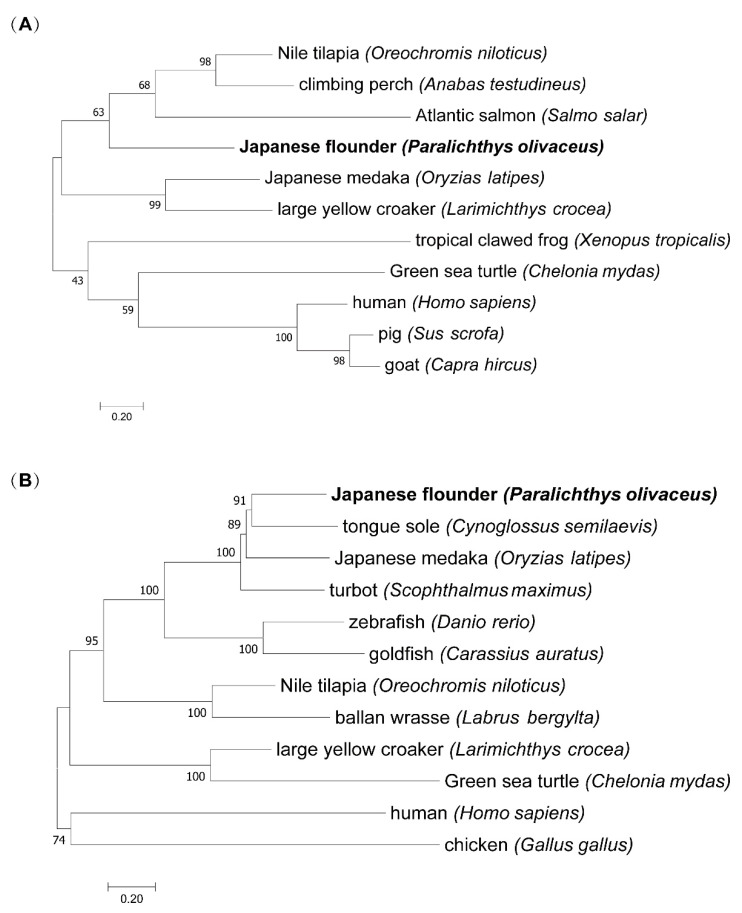
(**A**) *Bad* phylogenetic analysis. The accession numbers of Bad protein are listed in Table S9. (**B**) *EPAS1* phylogenetic analysis. The accession numbers of EPAS1 protein are listed in Table S10. The numbers in the branches represent the percentage of the associated taxa clustered together in the bootstrap test (1000 replicates).

The evolutionary trees of *Bad* and *EPAS1* were constructed to understand the evolutionary relationship. The results showed that the phylogenetic tree of *Bad* was divided into two branches, fish and other advanced vertebrates (Figure 3A). Similarly, the phylogenetic tree of *EPAS1* was mainly divided into two branches (Figure 3B).

### 3.4. EPAS1 and Bad Gene mRNA Relative Expression

**Figure 4:**
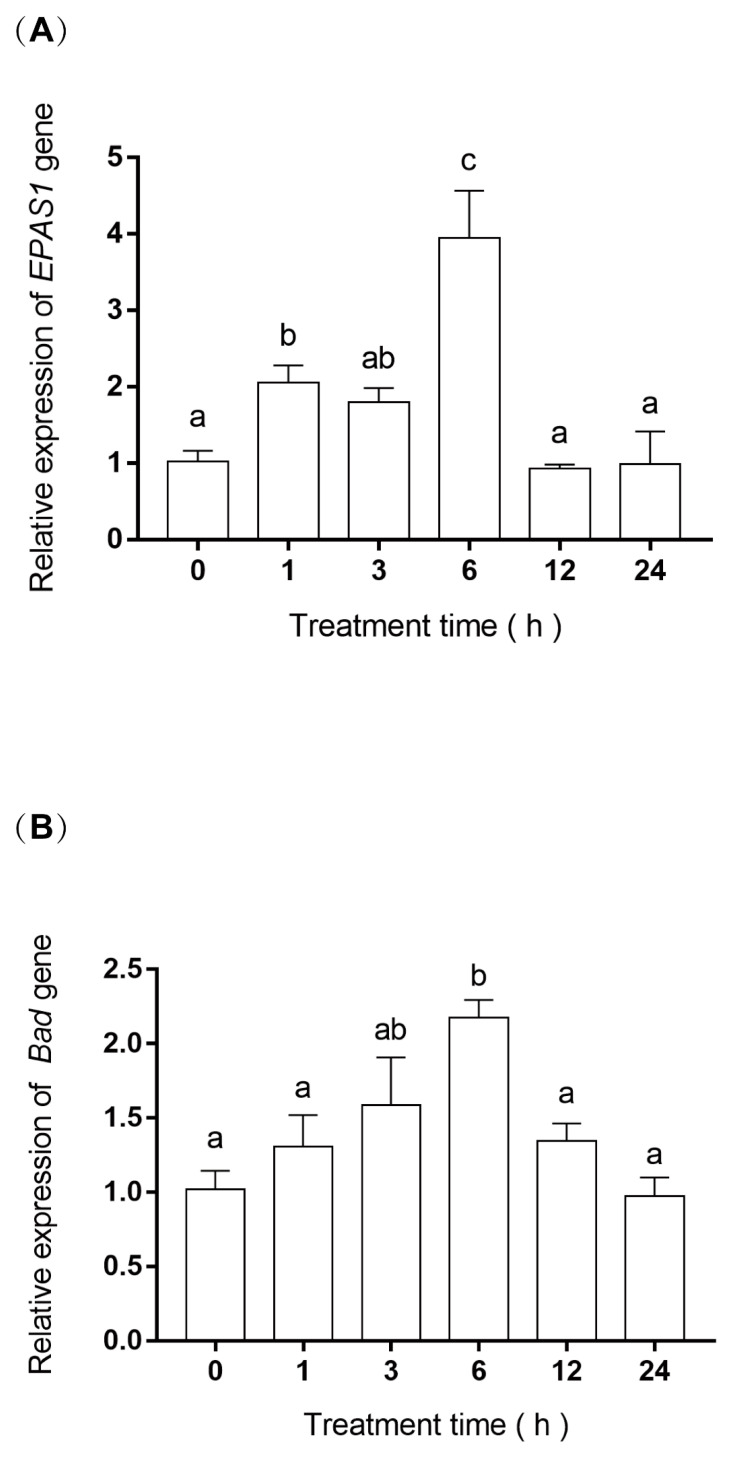
(**A**) The mRNA relative expression of *EPAS1* in gill. (**B**) The mRNA relative expression of *Bad* in gills. Significance was set at *p* < 0.05, and significant post hoc differences between time exposure are shown by uncommon letters.

The results of relative gene expression showed that *EPAS1* and *Bad* mRNA first increased and then decreased (Figure 4A,B). Specifically, *EPAS1* expression was about two-fold (*p* = 0.01) and four-fold (*p* = 0.00) higher at 1 h and 6 h of hypoxia than at 0 h, respectively, then decreased to near 0 h level. The mRNA expression level of *Bad* was similar to *EPAS1*, which also increased about two-fold (*p* = 0.00) at 6 h of hypoxia, and then decreased gradually.

### 3.5. EPAS1 Transcriptionally Regulated Bad

**Figure 5:**
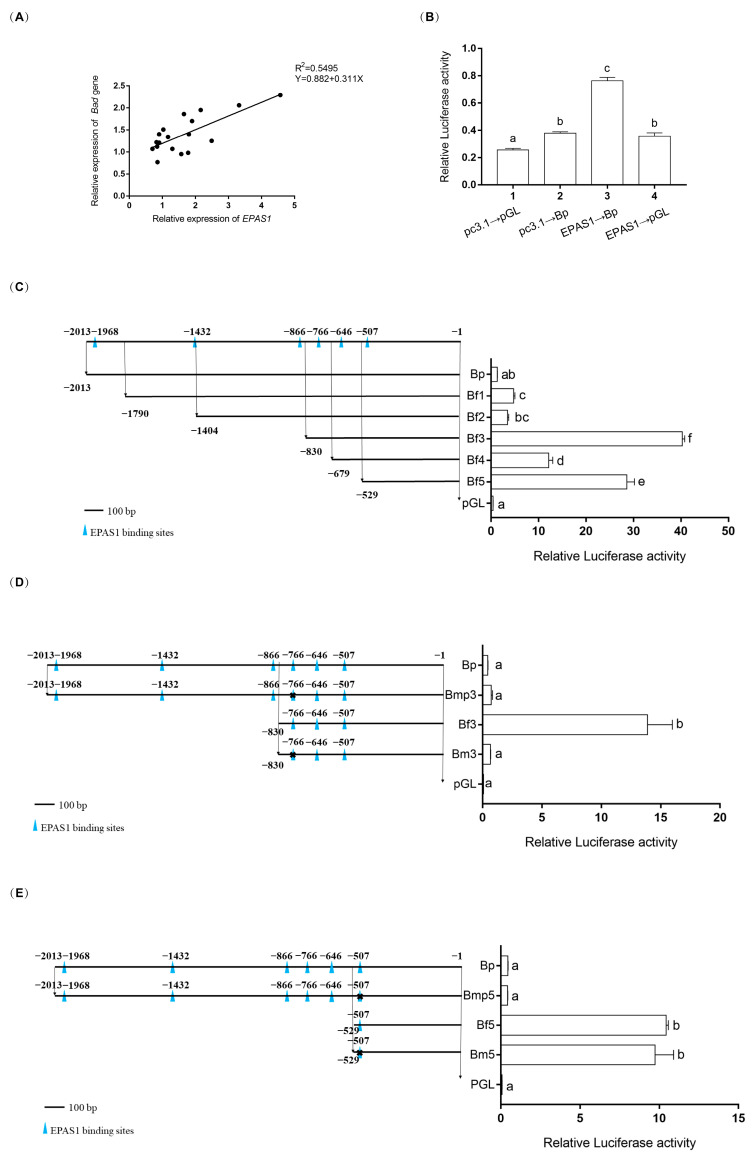
Regulation of transcription factor *EPAS1* on *Bad* promoter by dual-luciferase reporter assay. (**A**) The relationships of mRNA relative expressions between *EPAS1* and *Bad*. (**B**) Regulation of transcription factor *EPAS1* on *Bad* promoter. The pGL represented circular pGL3-Basic plasmid and pc3.1 represented circular pcDNA3.1(+) plasmid. *EPAS1* represented expression plasmid pc3.1∼*EPAS1* while Bp represented reporter plasmid pGL∼*Bad*. (**C**) The fragmentation deletion result. The blue triangle icons (▲) represented the putative *EPAS1* binding sites. The six light gray horizontal lines (Bp, Bf1, Bf2, Bf3, Bf4, Bf5) represented plasmids (pGL~*Bad*, pGL~Bf1, pGL~Bf2, pGL~Bf3, pGL~Bf4, pGL~Bf5), which were linked by promoter sequences of *Bad* gene with different lengths and fragmented pGL3-Basic digested by double endonuclease (KpnI and HindIII), respectively. Bp plasmids and pGL plasmids were similar to pGL∼*Bad* and pGL3, respectively, in (**B**). The numbers on the light gray horizontal lines represent the relative position of the binding sites in the *Bad* promoter sequence. (**D**,**E**) The bases mutation result. Bmp3 and Bm3 represented the two mutant plasmids (pGL~Bmp3, pGL~Bm3) with deleted putative *EPAS1* binding sequence (AATGGAAAC) near −766 (D). Bmp5 and Bm5 represented the two mutant plasmids (pGL~Bmp5, pGL~Bm5) with deleted putative *EPAS1* binding sequence (TCTGTGTGTCAG) near −507. (**E**). Significance was set at *p* < 0.05, and significant post hoc differences between different groups are shown by uncommon letters.

There is a positive correlation (R = 0.741, R^2^ = 0.5495, Figure 5A) between the mRNA relative expressions of *EPAS1* and *Bad* using the linear regression analysis. As shown in Figure 5B, the relative luciferase activities were upregulated about two-fold (*p* = 0.00) when the cotransfection pGL3~*Bad* and pc3.1~*EPAS1* into HEK293T cells. The assay of luciferase activity showed that the transcription of pGL3~*Bad* was upregulated by pc3.1~*EPAS1*. The method of fragment deletion was used to detect the specific binding sites of transcription factor *EPAS1* (Figure 5C). The results showed that the first significant (*p* < 0.05) difference occurred between Bf3 and Bf4, and the fluorescence value of Bf3 is about 99-fold higher than that of the pGL group. The second significant (*p* < 0.05) difference occurred between Bf5 and pGL and the fluorescence value of Bf5 is about 70-fold higher than that of the pGL group. These results indicated that the EPAS1 binding sites were located between −830~−679 or −529~−1. Therefore, the two predicted binding sequences were mutated. The relative luciferase activity of the first mutant plasmid was about 21-fold lower (*p* = 0.00) than that of the Bf3 group (Figure 5D). However, the relative luciferase activity of the second mutant plasmid was not obviously different from that of the control plasmid (Figure 5E).

### 3.6. DNA Methylation Levels of EPAS1 and Bad Gene

**Figure 6:**
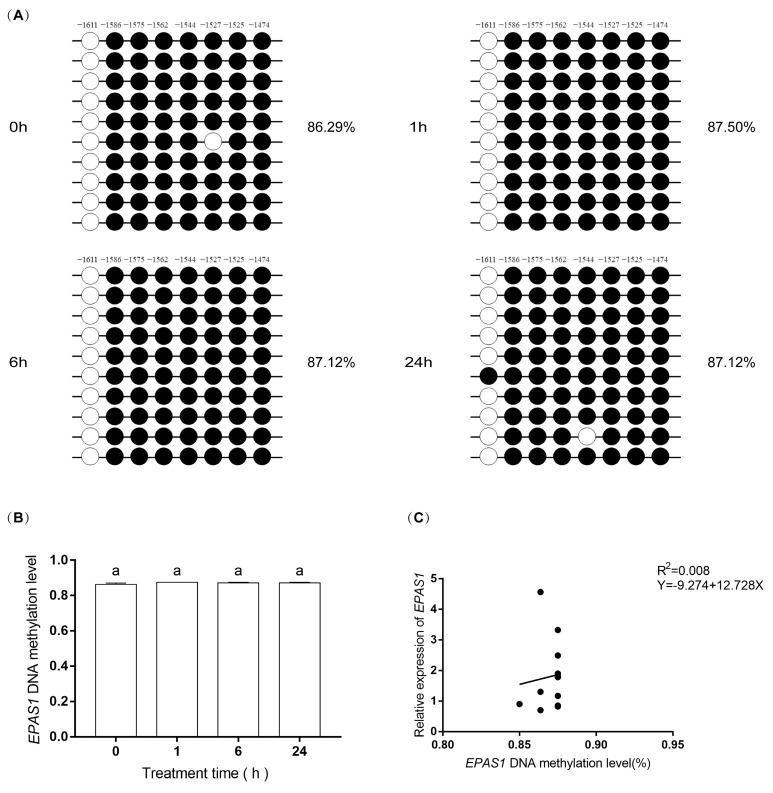
The methylation level of *EPAS1* gene promoter at 0 h, 1 h, 6 h, and 24 h after hypoxia. (**A**) The methylation status and levels of *EPAS1* gene. The circles show the methylation status. The numbers above the circles represent 8 CpG dinucleotides. The percentage on the right of the circles is the average methylation level of the corresponding group. (**B**) The methylated levels of *EPAS1* gene. The three biological replicates methylation levels in the 1 h group were consistent, so there was no visible SE. (**C**) Linear regression analysis of methylation levels and mRNA relative expression levels of EPAS1.

The methylation status of *EPAS1* promoter and *Bad* promoter are shown in Figure S3. There are 8 CpG dinucleotides per 193 bp in *EPAS1*. The results showed that the total methylation levels were 87.01% ± 0.22% (Figure 6A,B). Linear regression analysis was conducted between methylation levels and mRNA expression levels of *EPAS1*, but there was no relationship between them (R = 0.088, R^2^ = 0.008; Figure 6C).

A black circle (●) indicates methylation and a hollow circle (○) indicates unmethylation. The numbers above represent 8 CpG dinucleotides, and their sites are the distance from the starting codon. These sites are −1611, −1586, −1575, −1562, −1544, −1527, −1525, and −1474. The same letter, “a” indicated non-significant differences (*p > 0.05*).

**Figure 7:**
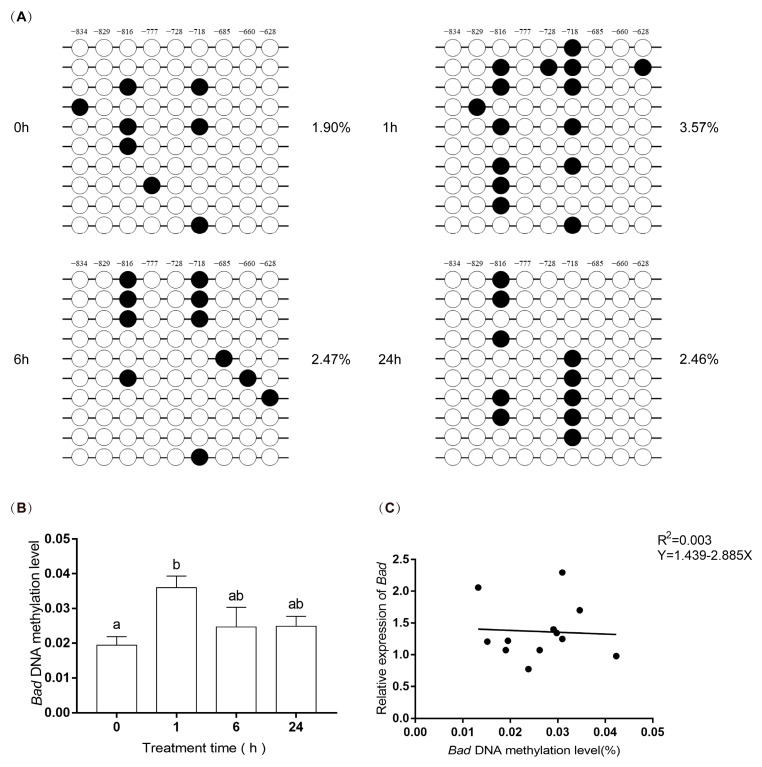
The methylation level of *Bad* gene promoter at 0 h, 1 h, 6 h, and 24 h after hypoxia. (**A**) The methylation status and levels of *Bad* gene. The circles show the methylation status. The numbers above the circles represent 9 CpG dinucleotides. The percentage on the right of the circles is the average methylation level of the corresponding group. (**B**) The methylated levels of *Bad* gene. (**C**) Linear regression analysis of methylation levels and mRNA relative expression levels of *Bad*.

There are 42 CpG dinucleotides per 400 bp in *Bad*. The total methylation level of the *Bad* gene displayed a lower level (2.62% ± 0.23%) compared to the *EPAS1* gene. Compared with the 0 h group, the change trends first increased about two-fold (*p* = 0.01) at 1 h after hypoxia and then gradually decreased with the prolongation of hypoxia stress (Figure 7A,B). This methylation change was mainly concentrated at the 19th (−816 site) and 30th (−718 site) CpG sites. Furthermore, linear regression analysis was conducted between methylation levels and mRNA relative expression levels of *Bad*, but there was no relationship between them (R = 0.055, R^2^ = 0.003) (Figure 7C).

A black circle (●) indicates methylation, and a hollow circle (○) indicates unmethylation. There are 42 CpG sites, and these sites are the distance from the starting codon. These sites are −939, −928, −926, −921, −910, −901, −893, −891, −872, −856, −845, −839, −837, −834, −832, −829, −824, −818, −816, −814, −811, −799, −797, −777, −774, −755, −730, −728, −723, −718, −712, −696, −685, −673, −660, −640, −630, −628, −608, −603, −599, −594. Nine sites are marked in Figure 7A: −834, −829, −816, −777, −728, −718, −685, −660, −628. Significance was set at *p* < 0.05, and significant post hoc differences between time exposure are shown by uncommon letters.

## 4. Discussion

Hypoxia is a serious problem in aquaculture, which affects the survival of aquatic animals. Hypoxia exposure caused apoptosis of ovarian follicle cells in Atlantic croaker [32], the central system of the sturgeon of sturgeon (*Acipenser shrenckii*) [33], skeletal muscle in Japanese flounder(*Paralichthys olivaceus*) [27], and gills in blunt snout bream (*Megalobrama amblycephala*) [8]. In all these studies, hypoxia seriously affected fish survival and development and caused severe apoptosis. In this study, the gills of Japanese flounder were curved at 6 h and 24 h of hypoxia, and the ILCM volume decreased after hypoxia, which increased the contact area between gill lamellae and water. At the same time, the TUNEL apoptosis signals almost began to increase with hypoxia, and the signals were very dense at 6 h of hypoxia. However, there were few TUNEL apoptotic signals in the 0 h group. These results indicated that Japanese flounder would have a significant apoptotic response to acute hypoxia stress, with a reduction of the ILCM and elongation of gill lamellae, which indicates the expansion of the respiratory surface area to adapt to a more severe hypoxia environment.

*Bad* is very essential for apoptosis [34]. We analyzed the mRNA expression of the *Bad* gene in the gills of Japanese flounder under hypoxic stress, the results indicated that the expression level of *Bad* was upregulated about two-fold after 6 h of hypoxia compared with 0 h of control. Therefore, the upregulated expression of *Bad* may influence the apoptosis of Japanese flounder gills against hypoxic stress. Similar to our study, the expression of *Bad* in blunt snout bream was significantly upregulated after 4 and 7 days of hypoxia, and the apoptosis rate of gill cells was also significantly increased [8]. Especially, *EPAS1* showed similar changes in gene expression that the expression level was first increased about four-fold at 6 h after hypoxia and then decreased. Meanwhile, the expression of *EPAS1* and *Bad* genes was analyzed by linear regression, and the results showed that their expressions were positively correlated. Their similar gene expression suggests that they may be important in hypoxia-induced apoptosis. Studies have shown that the gills of crucian carp showed protruding lamella during hypoxia, which may be caused by the combination of reduced cell proliferation and increased apoptosis induction. Therefore, the increased expression of the *Bad* gene may lead to an increase in apoptosis, which has been confirmed in the TUNEL experiment. These results may be the reason for the increase in gill respiratory surface area.

Hypoxia may cause apoptosis. *HIF-1α*, a relative of *HIF-2α/EPAS1*, could activate the expression of *Nip3* (member of the Bcl-2 family) in response to hypoxia because of the functional HIF-1-responsive element (HRE) in the *Nip3* promoter [35]. It is speculated that *EPAS1* may be directly related to the promoter of *Bad*. Here, the results of the dual-luciferase reporter assay indicated that *Bad* is regulated by *EPAS1* (Figure 5B). EPAS1 could activate the *Bad* gene transcription. Dual-luciferase reporter assay was used to determine the specific binding sites of EPAS1 on the *Bad* gene promoter. According to the fragment deletion results in Figure 5C, the EPAS1 binding sites were likely around −766 or −507. When the above sites were mutated, the fluorescence value of the first site decreased about 21-fold, while the fluorescence value of the second site was almost unchanged (Figure 5D,E). Based on the results of the dual-luciferase reporter assay, we concluded that the binding site of EPAS1 was a sequence (AATGGAAAC) located near −766.

DNA methylation is essential for mammalian epigenetics [36]. Hypoxia can affect gene DNA methylation status. In the tumor, hypoxia acts as a regulator of DNA methylation [37]. Therefore, methylation-specific PCR [38] was used to detect the methylation status of *EPAS1* and *Bad* gene promoters. However, the promoter of *the EPAS1* gene was highly methylated at each treatment time. However, there was no relationship between the methylation status of *EPAS1* and its expression (Figure 6C). In addition, no CpG island was found in the proximal promoter region of the *Bad* gene, but there was a CpG island with 42 CG sites in the 5′noncoding region. The results of *Bad* methylation showed that hypoxia increased about two-fold in 1 h (Figure 7B), indicating that hypoxia could increase the methylation level of the *Bad* 5′non-coding region. However, there was no correlation between this methylation and *Bad* expression (Figure 7C). We speculated that the region currently detected is not an important site for DNA methylation, and the sites regulating the expression of *EPAS1,* and *Bad* genes may exist in the undetected CG sites [39]. In other words, the upregulation of *EPAS1* and *Bad* genes may be related to the methylation degree of other regions of its promoter. Secondly, *EPAS1* and *Bad* genes may be regulated by other genes on hypoxia-related pathways. These results suggest that the methylation of *EPAS1* and *Bad* might not directly regulate the expression of their genes.

Hypoxia, as a cell death stimulus, caused apoptosis of gill in Japanese flounder. In response to hypoxic stress, gill tissue structure rapidly changed, followed by rapid upregulation of *EPAS1* and *Bad* gene expression in response. Meanwhile, the apoptosis cells in the gill were increased after 6 h of hypoxia compared to 0 h of control. This experiment identified the binding site of *EPAS1* on the *Bad* promoter and attempted to solve the regulatory relationship of this signal pathway from the epigenetic modification. Collectively, *EPAS1/Bad* signaling pathways are important for hypoxia-induced apoptosis. These findings reported here shed new light on a direct link between *EPAS1* and a proapoptotic member of the Bcl-2 family and offer a reasonable apoptosis mechanism for hypoxia-stimulated Japanese flounder.

## 5. Conclusions

Acute hypoxia stress can activate the *EPAS1/Bad* signal pathway, by raising *EPAS1* expression to promote the expression of *Bad*, leading to apoptosis of Japanese flounder gill. This signal pathway may not be directly regulated by DNA methylation. In conclusion, hypoxia induced gill apoptosis in Japanese flounder by acting on the hypoxic signal pathway *EPAS1/Bad* in transcriptional regulation. This study enriched the mechanism of hypoxia-induced gill apoptosis in Japanese flounder and provided a new idea for healthy fish culture.

## Data Availability

Data used to support the findings from this study are available from the corresponding author upon request.

## Supplementary Materials

The following supporting information can be downloaded at: https://www.mdpi.com/article/10.3390/biology11111656/s1, Table S1: Primers used for q-PCR. Table S2: System used for q-PCR. Table S3: Procedure used for q-PCR. Table S4: System and procedure used for double digestion. Table S5: Primers of plasmid construction for dual-luciferase reporter assay. Table S6: System used for dual-luciferase reporter assay. Table S7: Procedure used for dual-luciferase reporter assay. Table S8: Primers of methylation status detecting in EPAS1 and Bad promoter by MS-PCR. Table S9: The accession numbers of Bad protein. Table S10: The accession numbers of EPAS1 protein. Figure S1: The melt curves of primers for three genes EPAS1 (A), Bad (B), 18S (C) in qPCR. Figure S2: The predicted binding site of EPAS1 on the Bad promoter. Figure S3: Methylation status of EPAS1 promoter and Bad promoter. (A) The methylation status measuring region of the EPAS1 gene. The abscissa indicates a part of the EPAS1 gene; ordinate denotes CG percentage; the blue area shows the CpG island (CG percentage more than 50%, −1626~−1349, 278 bp). (B) The 193 bp sequence of methylation status measurement in the EPAS1 gene. The 8 CpG dinucleotides are indicated in red letters. (C) The methylation status measuring region of the Bad gene. The abscissa indicates a part of the Bad gene; ordinate denotes CG percentage; the blue area shows the CpG island (CG percentage more than 50%, −953~−555, 399 bp). (D) The 400 bp sequence of methylation status measurement in the Bad gene. The 42 CpG dinucleotides are indicated in red letters.
