# Supplementary material for: Acute Hypoxia Stress-Induced Apoptosis in Gill of Japanese Flounder (Paralichthys olivaceus) by Modulating the Epas1/Bad Pathway
Source: Biology (Basel). 2022 Nov 12;11(11):1656. doi: 10.3390/biology11111656 (PMC9687431; doi:10.3390/biology11111656)
Supplement: Supplementary file 1 [file biology-11-01656-s001.zip › biology-1927202-supplementary.pdf]

Supplementary Materials

Table S1 Primers used for q-PCR.

Primers	Sequences (5′-3′)	Annealing temperature (℃)	Product size (bp)	Accession number
Bad	F:TCGGAGGAGATAGAGGAAGC<br>R:TCGTGAGACAGTGGAGGCGT	60℃	156 bp	XM_020095653.1 (accessed on 28 August 2022)
EPAS1	F:ATGAGTGGCACAAACCAGAAG<br>R:AGACAGGAGGGCTCGAACGAG	60℃	131 bp	XM_020096630.1 (accessed on 28 August 2022)
18S	F:ATTGACGGAAGGGCACCAC<br>R:ATGCACCACCACCCACAGA	60℃	134 bp	EF126037.1

Table S2 System used for q-PCR.

Component	Volume
2 × ChamQ SYBR Color qPCR Master Mix (High ROX Premixed)	10.0 μl
Forward Primer	0.4 μl
Reverse Primer	0.4 μl
cDNA	2.0 μl
ddH2O	7.2 μl

*The RNA concentration of the samples was 500ng/ul, and the concentration of cDNA obtained by reverse transcription of RNA was 1000ng/ul.

Table S3 Procedure used for q-PCR.

Stage	Repeat	Temperature	Time
Stage 1	Rep: 1	95 ℃	30 sec
Stage 2	Rep: 40	95 ℃	10 sec
		60 ℃	30 sec
Stage 3	Rep: 1	95 ℃	15 sec
		60 ℃	60 sec
		95 ℃	15 sec

Table S4 System and procedure used for double digestion.

Component	Volume
Restriction Enzyme (KpnI / HindIII / BamHI / Xhol)	1.0 µl
DNA	1.0 µg
10X NEBuffer	5 µl
ddH2O	To 50.0 µl
Incubation Time	3 hours
Incubation Temperature	37℃

Table S5 Primers of plasmid construction for dual-luciferase reporter assay.

Primers	Sequences (5′-3′)	Annealing temperature (℃)	Product size (bp)	Accession number
pc3.1~*EPAS1*	F:cttggtaccgagctcggatccCGTGCAGAGGAGGAAACAT<br>R:aacgggccctctagactcgagACACCAAAAGACCCGAGTT	54°C	2982 bp	XM_020096630.1 (accessed on 28 August 2022)
pGL~*Bad*	F:atttctctatcgataggtaccACGGTGGTTTTATGGCTTA<br>R:cagtaccggaatgccaagcttGGCTTGACTTTTCTGTGTG	51°C	2013 bp	109634873
pGL~Bf1	F:atttctctatcgataggtaccCGTCAGTGTATGAAAGGCT<br>R:cagtaccggaatgccaagcttGGCTTGACTTTTCTGTGTG	52°C	1790 bp	109634873
pGL~Bf2	F:atttctctatcgataggtaccTCAGGGGATGAGAAACACT<br>R:cagtaccggaatgccaagcttGGCTTGACTTTTCTGTGTG	52°C	1404 bp	109634873
pGL~Bf3	F:atttctctatcgataggtaccGGGCCTTAAAAAGCCTGAA<br>R:cagtaccggaatgccaagcttGGCTTGACTTTTCTGTGTG	52°C	830 bp	109634873
pGL~Bf4	F:atttctctatcgataggtaccGCCTCACCCCTACAAAAAA<br>R:cagtaccggaatgccaagcttGGCTTGACTTTTCTGTGTG	52°C	679 bp	109634873
pGL~Bf5	F:atttctctatcgataggtaccACGAGGTAGGTTGTTCTTC<br>R:cagtaccggaatgccaagcttGGCTTGACTTTTCTGTGTG	52°C	529 bp	109634873
pGL~Bmp3	F:TAAGTGCTTGGTTGAAACTGGCTGAATAAATCG<br>R:GTTTCAACCAAGCACTTATAAATTAGATGAATAA TATTAAAAATATTATAATCATTCAGG	53°C	2004 bp	109634873
pGL~Bm3	F:TAAGTGCTTGGTTGAAACTGGCTGAATAAATCG<br>R:GTTTCAACCAAGCACTTATAAATTAGATGAATAA TATTAAAAATATTATAATCATTCAGG	53°C	821 bp	109634873
pGL~Bmp5	F:TTCTTCATCTTTGATTCATTAATGGCAAGATGTAA CAAAG<br>R:TGAATCAAAGATGAAGAACAACCTACCTCGT	53℃	2001 bp	109634873
pGL~Bm5	F:TTCTTCATCTTTGATTCATTAATGGCAAGATGTAA CAAAG<br>R:TGAATCAAAGATGAAGAACAACCTACCTCGT	53°C	517 bp	109634873

Table S6 System used for dual-luciferase reporter assay.

Component	Volume
2 × Phanta Max Master Mix (Dye Plus)	25.0 µl
Forward Primer	2.0 µl
Reverse Primer	2.0 µl
cDNA (*EPAS1*) / DNA(*Bad*)	1.0 µl
ddH2O	20.0 µl

Table S7 Procedure used for dual-luciferase reporter assay.

Stage	Repeat	Temperature	Time
Stage 1	Rep: 1	95 ℃	3 min
Stage 2	Rep: 30	95 ℃	15 sec
		60 ℃*	15 sec
		72 ℃	30 - 60 sec/kb
Stage 3	Rep: 1	72 ℃	5 min

*Set annealing temperature according to melting temperature (Tm) value of primer.

Table S8 Primers of methylation status detecting in *EPAS1* and *Bad* promoter by MS-PCR.

Primers	Sequences (5′-3′)	Annealing temperature (℃)	Product size (bp)	Accession number
EPAS1 -M	F:ATTTTATAATGTAAGAAAAGTTGGTTGTGT	50°C	193bp	109635465
	R:TTAAATCATTTCAAAAACAAATCTC			
Bad -M	F:ATAGAAAAGTTAAGTTTGATTTGTGA	48.8°C	400bp	109634873
	R:ACTAAAAAAAACAAAAAAAACTTCCTA			

Table S9 The accession numbers of Bad protein.

Species	Accession numbers
human *(Homo sapiens)*	NP_004313.1
goat (*Capra hircus*)	XP_017898279.1
pig (*Sus scrofa*)	XP_020938542.1
Green sea turtle (*Chelonia mydas*)	XP_007072774.3
tropical clawed frog (*Xenopus tropicalis*)	XP_031756504.1
large yellow croaker (*Larimichthys crocea*)	XP_010741574.1
Atlantic salmon (*Salmo salar*)	XP_014051932.1
Japanese medaka (*Oryzias latipes*)	XP_011485704.1
Nile tilapia (*Oreochromis niloticus*)	XP_003452465.1
climbing perch (*Anabas testudineus*)	XP_026208848.1
Japanese flounder (*Paralichthys olivaceus*)	XP_019951212.1

Table S10 The accession numbers of EPAS1 protein.

Species	Accession numbers
Nile tilapia (Oreochromis niloticus)	XP_003438301.1
Japanese medaka (*Oryzias latipes*)	XP_023819367.1
large yellow croaker (*Larimichthys crocea*)	XP_019116438.1
ballan wrasse (*Labrus bergylta*)	XP_020511317.1
turbot (*Scophthalmus maximus*)	XP_035461545.2
Japanese flounder (*Paralichthys olivaceus*)	XP_019952189.1
tongue sole (*Cynoglossus semilaevis*)	XP_008320789.1
zebrafish (*Danio rerio*)	XP_695262.6
goldfish (*Carassius auratus*)	XP_026132160.1
chicken (*Gallus gallus*)	XP_046794384.1
Green sea turtle (*Chelonia mydas*)	XP_043399726.1
human (*Homo sapiens*)	XP_011531000.1

Figure S1 The melt curves of primers for three genes *EPAS1* (A), *Bad* (B), 18S (C) in qPCR.

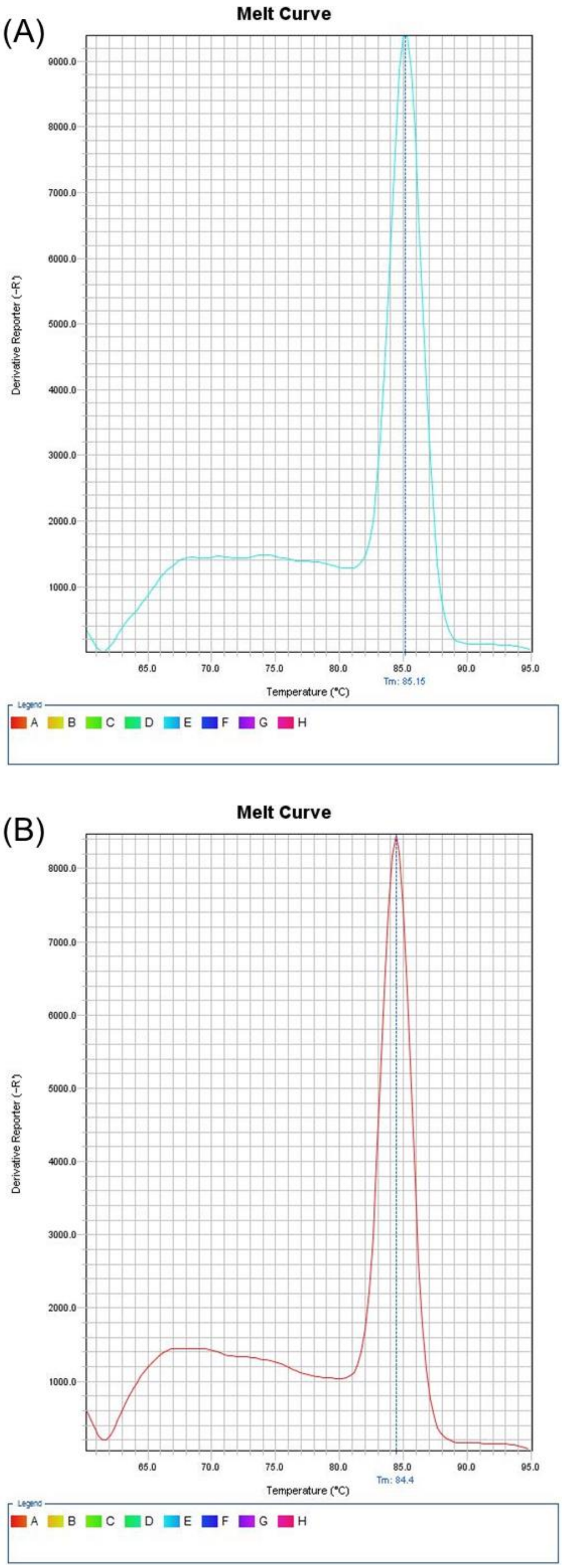

(C)

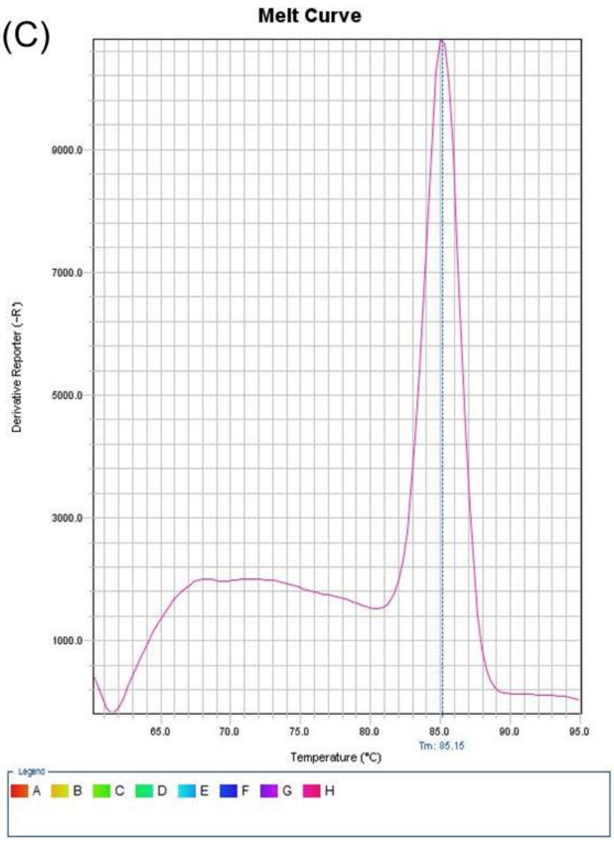
Melt Curve
Derivative Reporter (-R')
9000.0
7000.0
5000.0
3000.0
1000.0
65.0
70.0
75.0
80.0
85.0
90.0
95.0
Tm: 85.15
Temperature (°C)
Legend
A
B
C
D
E
F
G
H

Figure S2 The predicted binding site of EPAS1 on the *Bad* promoter.

-2013 **-1968 Binding site 1**
ACGGTGGTTTTATGGCTTAAGTTGGTGCATACATGGCTTGACTTTGCCACACACACACGCACGCACACACAGA
TTAAAATGGATAATAATTGCATGGTTCTCAAACTGCATCCAAATCCAACCAAAAGGAGAACTCCAGTGTATT
ATCTCTATAATCCCTCCATAAATACAGTTCAAAAGAAGCAACTCTCAACAATGATCTTCAAGAGCTGCTTCAT
GCATACGTCAGTGTATGAAAGGCTGACTGTGCAAACTTTGCACTACATGTTAGTCTAAGCTTAGAAACTCACA
TTTGTTCCATTTGGATGCACTGAATTAGAAAAAGCCCTTTGACATTGAATACATCACAGCATTGTTTCAATGA
CTGACTTTGCCAAATTAATTCAGTTTATTTCTAGTGTCTAATATAATAGGGGGAGAGAGCAGAATGACATTGG
GGAGGTGAAGAACAGGCTCTGGATTAAAAATGTTAGATAATTACATGGCTGAGTCTCCTGTGTTGACGTGAG
GAAAGTGCAAGAGAGCAAAGCAAAGCACATACCCATACAGTGCTGCTTTACTGTAACGGTTCTGTATAAAGA
-1432 Binding site 2
AGAGGAATGTAACCCATTAATCAGCTTTTCAGGGGATGAGAAACACTTTTTTCTGATGATGTCACAATGCAAT
TTTCATGCCTGACACTATGACACGTTTTTGCAGCAATAATGCTACTAGAGTAATCAACTGCAATTGAAAGACA
TTCAAAATAAGCTATTTGCTCGTATTGTTTCCAAGAAAGCTCACAACCTGTGACAGGAGGTAGGAGATGAGG
CAGTGCGACCAAAGATAAACACTCAGTAAAACACACACACAAGTGAATCAGTTTGTAGTCCTACCTCCCCAC
CTGCTGCTCATGCGAGGTATGACAGCTAACAACAGCCTGGAGGATTTTCTGTCTTCCATGTGTCCCAGCCATA
GGGTCCCAGCACTAGAGAAAGCAGAGAAGATAAAAGAAGAGCTTAACCTTAAGACCTTCGAGGAACTGAAT
GTTAATGTGATTCCTCCTCGATATGAATCAATTAAAAATAACTTTACCGGATGATTATTACATGTCATGTAAT
-866 Binding site 3
GACAAGAGCAAATCTCACATAAACATTTATCACAGAAACATTATCATAGTGGGGGGGAAGAGTGGAACGGA
CTTCCAAAGGCCCAAAGGGGAGGGGGGCCTTAAAAAGCCTGAATGATTATAATATTTTTAATATTATTCATCT
-766 Binding site 4
AATTTATAAGTGCTTAATGGAAACGGTTGAAACTGGCTGAATAAATCGACTGATTGACTGAAATTTATATCTA
-646 Binding site 5
AAAATCGTAGAGGCACCCTGAGGTCACCTGCCTCACCCCTACAAAAAAACGGTTTAGTCGAAGTGACTGACT
GACTACTGGAGAAGTGGTGTTCAGCATGTCAAGAAAGCAAACGGAAAGTCTGGGAATCAGAAAATAAAAAA
-507 Binding site 6
TATAAAAAATAAATACTAGTTAGTTAGTTGGTTCTTACGAGGTAGGTTGTTCTTCATCTCTGTGTGTCAGTTTG
ATTCATTAATGGCAAGATGTAACAAAGTGAGAGATTACACCCCATTTATAAATGAATACTTAATGGGAGGAA
AGCTGTATTTTAAAAATGACTTAGGGGGTTAGAAAACTAAGATGGTATTTTTAAGGTTTGTTTTATACTTGAG
AAATAATAAAGTAACTGTAGGTAAAAGTCTGAGAAAGGAGGACAGAAATGCAGCGAGATTTCATATTGTGC
ATAATTAAGCATATCAGATCATTATTACTTATACCAGATGTGGTATGTACTGTTCATTAGTGAGACATGGGGG
CCCACAGATACTGCTTGTGTATAGGGCCCAGAATCTTGTGCTACGCCCCCTGGTGGTGAGTAGATAATGAGTG
AATGTTCATGTTTTGGGTGAATTATCCCTTCAATTTGCAGTTGAGTGAATTTGGCTTCACTTTTGGGGATCTGT
CATATTTATATATCAATCATTATATCAATTCTACCACACACAGAAAAGTCAAGCC **-1**

The bases under the blue shadow are the predicted binding site sequence.

Figure S3 Methylation status of *EPAS1* promoter and *Bad* promoter.

(**A**)

EPAS1

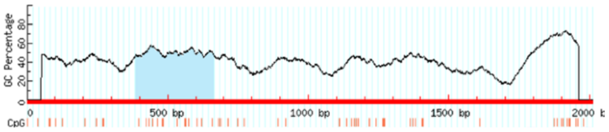

(**B**)

ACCTTACAATGTAAGAAAAGTTGGTTG
TGCTCGGGTGGTTCCTCCTCTGGTGCTG
CCGCTCTGTAGCCGACTGATCTTTTCG
ATCTAAAACTCCTGAGCGGTTGACAGG
AGAGGACGCGGTGAAATGCCAGAAGC
AGTTATCTGTCTGTTGGTTTGTGAAAA
ATGTTGCGAGACCTGCCCCTGAAATGA
CTTA

(**C**)

Bad

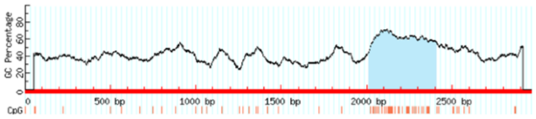

(**D**)

ACAGAAAAGTCAAGCCTGACTTGTGAC
GGTGTGAAATCGCGCTGCGCCTCTACC
CCGCACACTACGGTCACCCGCGGGGGA
CAAACAAAGCCCCGGCCCTGTCCTAGC
TCGCAACAGTGACGTCAGCGCGCCGCG
GCGTTGCGGTGACGCGCGGCGATTGGG
TGAGCGCGGAGCTAAAGTGAGCAGCTC
GGCGAGGAAAAGATAAGACAGCGACA
ATAACAGCAGCTGCAGAGGCCGCGGG
ACGCTCCGCCAACGCAAGCCCACACTC
CCGGACTCTCACCGCACATCAACTCGG
GTAAGAGACCCGGTGTGAAGGTTAGTT
CTTCGGTCCCTGACGCGGTTAGCTAGCA
GCCAAGCCGCAGCGGCCGCCCCGCAGG
AAGTTTCTTTTGTCCTCCTCAGT

(A) The methylation status measuring region of the *EPAS1* gene. The abscissa indicates a part of the *EPAS1* gene; ordinate denotes CG percentage; the blue area shows the CpG island (CG percentage more than 50%, −1626~−1349, 278 bp). (B) The 193 bp sequence of methylation status measurement in the *EPAS1* gene. The 8 CpG dinucleotides are indicated in red letters. (C) The methylation status measuring region of the *Bad* gene. The abscissa indicates a part of the *Bad* gene; ordinate denotes CG percentage; the blue area shows the CpG island (CG percentage more than 50%, -953~-555, 399 bp). (D) The 400 bp sequence of methylation status measurement in the *Bad* gene. The 42 CpG dinucleotides are indicated in red letters.
